# XUV excitation followed by ultrafast non-adiabatic relaxation in PAH molecules as a femto-astrochemistry experiment

**DOI:** 10.1038/ncomms8909

**Published:** 2015-08-13

**Authors:** A. Marciniak, V. Despré, T. Barillot, A. Rouzée, M.C.E. Galbraith, J. Klei, C.-H. Yang, C.T.L. Smeenk, V. Loriot, S. Nagaprasad Reddy, A.G.G.M. Tielens, S. Mahapatra, A. I. Kuleff, M.J.J. Vrakking, F. Lépine

**Affiliations:** 1 Institut Lumière Matière, Université Lyon 1, CNRS, UMR 5306, 10 rue Ada Byron, 69622 Villeurbanne Cedex, France; 2 Max-Born-Institut, Max Born Strasse 2A, D-12489 Berlin, Germany; 3 Leiden Observatory, Leiden University, PO Box 9513, NL-2300RA Leiden, The Netherlands; 4 School of Chemistry, University of Hyderabad, Hyderabad 500 046, India; 5 Theoretische Chemie, PCI, Universität Heidelberg, Im Neuenheimer Feld 229, D-69120 Heidelberg, Germany

## Abstract

Highly excited molecular species are at play in the chemistry of interstellar media and are involved in the creation of radiation damage in a biological tissue. Recently developed ultrashort extreme ultraviolet light sources offer the high excitation energies and ultrafast time-resolution required for probing the dynamics of highly excited molecular states on femtosecond (fs) (1 fs=10^−15^s) and even attosecond (as) (1 as=10^−18^ s) timescales. Here we show that polycyclic aromatic hydrocarbons (PAHs) undergo ultrafast relaxation on a few tens of femtoseconds timescales, involving an interplay between the electronic and vibrational degrees of freedom. Our work reveals a general property of excited radical PAHs that can help to elucidate the assignment of diffuse interstellar absorption bands in astrochemistry, and provides a benchmark for the manner in which coupled electronic and nuclear dynamics determines reaction pathways in large molecules following extreme ultraviolet excitation.

## Abstract (web summary)

Extreme UV light sources allow us to study the dynamics of excited molecular stets over remarkably short timeframes. Here, the authors probe polyaromatic hydrocarbons—large organic molecules—and show their electronic excitation and subsequent ultrafast relaxation.

## Graphical abstract

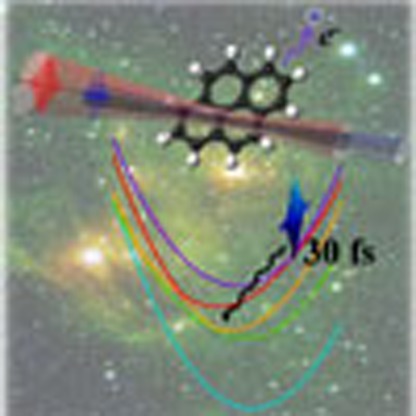

When a molecule interacts with a single UV or vacuum ultraviolet (VUV) photon, it can respond by promoting one of its electrons to an excited bound state or to the ionization continuum, leaving a hole in the electron density. At its simplest, this excitation process can be theoretically described using a single-particle picture. While such independent electron approximations are often sufficient in low-frequency light fields, they rapidly fail when excitation at higher photon energies (that is, in the extreme ultraviolet (XUV) domain) is considered. In this case, electron correlation cannot be neglected, and multi-electronic effects in the ionization are important, leading to processes such as Auger decay and shake-up/shake-off, where several electrons are ionized or excited as a result of the absorption of a single photon^1^.

Nuclear dynamics can occur following an electronic excitation. The typical case considered in femtochemistry experiments involves the excitation of single, well-defined electronic states that display time-dependent structural dynamics. This situation applies when the timescale for electronic motion (given by the inverse electronic level spacing) is much faster than that of the nuclear motion, and allows the use of the well-known Born–Oppenheimer approximation. In the Born–Oppenheimer approximation, the electrons instantaneously adapt to the nuclear geometry and the atoms move under the influence of forces that derive from potential energy surfaces describing the electronic energy as a function of nuclear geometry. The Born–Oppenheimer approximation breaks down when the timescales of the electronic and nuclear motion become comparable. In this case, the electronic and nuclear motion are strongly coupled, leading to ultrafast non-adiabatic electronic relaxation that typically takes place on a (sub)-picosecond time scale^2^.

The breakdown of both independent electron picture and Born–Oppenheimer picture upon excitation with high-energy photons was revealed in recent real-time investigations in simple molecules such as N_2_ (ref. 3), O_2_ (ref. 4) and N_2_O (ref. 5). The possible occurrence of multi-electronic mechanisms is even more pertinent in complex molecular species such as proteins, where they determine the production of slow electrons that are responsible for bond breaking, that is, radiation damage^6^,^7^. Likewise, non-Born–Oppenheimer effects at conical intersections (CIs) are prevalent in larger molecules, and are thought to be responsible for the photostability of DNA^8^. Understanding multi-electronic and non-adiabatic processes in large species at a microscopic level by means of time-resolved investigations is a major challenge within current ultrafast photochemistry research, made difficult by the rapidly increasing number of reaction pathways that occur when the size of the molecule increases.

In this article, we have investigated polycyclic aromatic hydrocarbon (PAH) molecules that are well known in astrochemistry, as they account for 10% of the elemental carbon in galaxies. Their photochemical fate is key to understand the origin of biologically relevant molecules, eventually leading to the emergence of life in the universe^9^. Interstellar large cationic PAHs (composed of several 10 C-atoms) are often considered to be prime candidates for being the carriers of the diffuse interstellar absorption bands (DIBs)—a set of some 400 absorption bands generally ascribed to (hitherto unidentified) interstellar molecules. Experimentally measured line widths of visible absorption bands of small PAH cations have been interpreted in terms of non-radiative relaxation pathways governed by an energy gap law^9^. This energy gap law has been taken to imply that for high-lying states and for large PAHs, where the density of electronic states is expected to be very high, the width of (visible) absorption lines becomes very large and incompatible with those observed for the DIBs. The linewidths of the DIBs imply that the lifetimes of the involved electronic states typically lie in the range of a few 10 s of fs to a few ps. Consequently, only the lowest electronic transitions are deemed to be relevant candidates for DIBs^10^, but the intensity of these transitions is too weak to support this hypothesis. As a result, the question of the origin of the DIBs remains puzzling. A definitive assignment of the DIBs to PAH molecules would be crucial as they illustrate the chemical complexity of the ‘molecular universe' (ref. 11 and references therein). In that context, quantitative determination of lifetimes of both small and larger, highly excited PAH cationic species is compulsory.

PAH molecules are also archetypal systems for which XUV-induced multi-electronic effects have been described^12^. Compared with more fragile molecules of the same size, they have the advantage of being rather chemically stable in the XUV spectral region. Therefore, from a fundamental point of view, they provide a situation where ionization involving many electrons, vibronic couplings and energy relaxation can be studied using high-level theoretical descriptions that need not take fragmentation into account^13^.

In this paper, we present the first experimental evidence for a general ultrafast mechanism that follows the absorption of an XUV photon by PAH molecules. This mechanism is sketched in Fig. 1a. An XUV photon ionizes the molecule creating a hole in the valence shell. As a result of electron correlation, the exit of the photoelectron may lead to excitation of another electron, that is, the formation of an excited ‘shake-up' state. The excited molecular cationic state relaxes through a non-adiabatic mechanism. The goal of our work is to unravel this mechanism and to determine its timescale, and thereby to establish the typical relaxation times of the excited radical PAHs.

## Results

### Experiments

**Figure 1:**
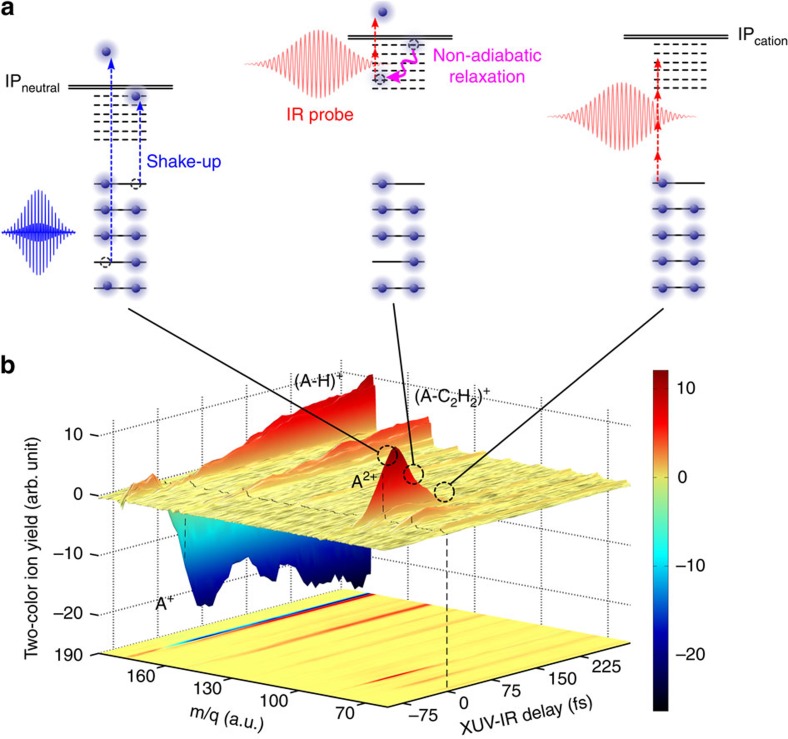
Schematic of the experiment. (**a**) Schematic of the XUV-induced dynamics in PAH molecules studied in this paper. Excited states are created in the valence shell of the cation through one of two possibilities, namely the formation of a single-hole configuration or the formation of a 2hole-1particle configuration (involving a shake-up process) (left) (IP stands for Ionization potential). The cation can be further ionized by the IR probe laser, provided that non-adiabatic relaxation has not taken place yet (middle). After relaxation, the IR probe cannot ionize the cation anymore (right). (**b**) Two-colour XUV+IR ion signals measured in the case of anthracene, as a function of the detected mass-to-charge ratio and the XUV-IR delay. XUV-only and IR-only signals have been subtracted. The XUV pump and IR probe pulses overlap at zero delay (black dashed line). A red colour corresponds to a signal increase, while a blue colour signifies depletion. For positive XUV-IR delays, a very fast dynamics is observed for the doubly charged anthracene ion (A^2+^, m/q=89). As explained in the text, the measurement reflects non-adiabatic relaxation in the anthracene cation (A^+^). The dynamics observed in the first fragment (A-C_2_H_2_^+^) is not discussed in this article.

The mechanism was experimentally demonstrated using a fs XUV pump—fs IR (infrared) probe arrangement (Supplementary Figs 1 and 2, and Supplementary Note 1). A 25-fs long XUV pulse, containing photon energies between 17 and 35 eV, ionized neutral PAH molecules, and was followed at a variable time delay by a 35-fs long, weak (typically 10^11^ W cm^−2^) IR pulse that probed the relaxation dynamics. The probing used ionization of the excited cationic states, and exploited the fact that rapid energy relaxation increases the energy that needs to be absorbed to doubly ionize the molecule, thus removing the molecules from the probe laser observation window. Experimentally, the formation of doubly charged cations was measured as a function of the XUV-IR pump-probe delay, as shown in Fig. 1b, where, for the case of anthracene, a 2-colour signal (ΔS) is shown, which is defined as


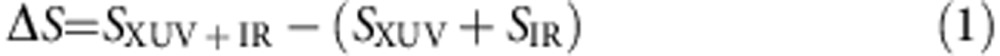


where *S*_XUV+IR_ corresponds to the ionization signal measured when both the XUV and IR lasers are present, and *S*_XUV_ and *S*_IR_ correspond to the signals measured when only the XUV laser or the IR laser present, respectively. As the main finding of our work, a rapid decay of the doubly charged parent ion signals was observed as a function of XUV-IR delay, for all four PAH molecules that were investigated: naphthalene (N, C_10_H_8_), anthracene (A, C_14_H_10_), pyrene (P, C_16_H_10_) and tetracene (T, C_18_H_12_).

**Figure 2:**
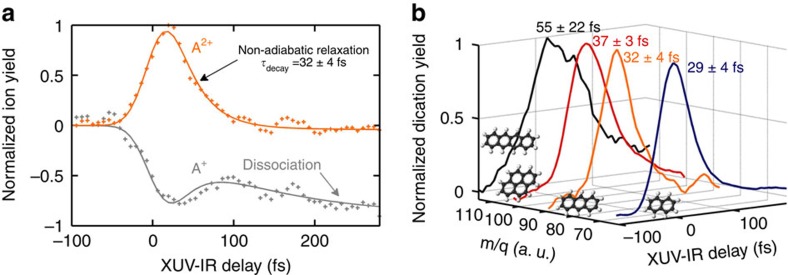
Time-dependent measurements. (**a**) Experimental time-dependent di-cationic (A^2+^, orange) and cationic (A^+^, grey) signals measured in XUV pump-IR probe experiments on anthracene (see Supplementary Note 2 for details about curve fitting). (**b**) Time-dependent di-cation signals measured for four PAH molecules: naphthalene (blue), anthracene (orange), pyrene (red) and tetracene (black).

To extract lifetimes from our measurements, the time-dependent doubly charged ion signals presented in Fig. 1b were fitted to an exponential decay convoluted with a Gaussian (see Fig. 2a):


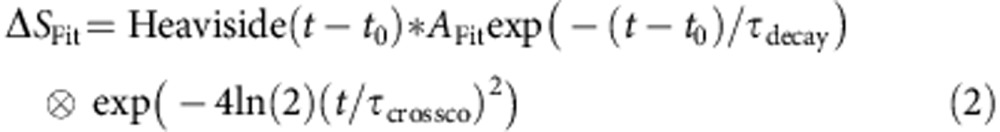


where *A*_Fit_ is the amplitude of the signal, *t*_0_ defines the time overlap of the XUV and IR pulses, *τ*_decay_ is the lifetime of the excited state, and *τ*_crossco_ is the full-width at half-maximum of a Gaussian function that accounts for the cross-correlation time between the XUV and IR pulses. In Fig. 2a results are shown for A^2+^, yielding an excited state lifetime of 32±4 fs. At delays >100 fs, the two-colour signal completely vanishes, since the IR intensity is no longer sufficient to ionize the cation A^+^. (For further details about the data analysis, see the Supplementary Note 2.)

In Fig. 2a the yield of the parent ion A^+^ is also plotted as a function of the XUV-IR pump–probe delay. For short XUV-IR delays (<100 fs), the time dependence of the A^+^ parent ion yield mirrors that of the A^2+^ doubly charged ion. This is consistent with our interpretation of the A^2+^ formation in terms of an IR-induced depopulation of excited cationic states, producing an A^2+^ ion and inhibiting the detection of an A^+^ ion. In other words, unless ionized by the IR laser, the XUV-induced excited states relax through internal conversion within a few tens of fs. At later delays a slow (>100 fs) reduction of the A^+^ yield appears, which reflects the occurrence of IR-induced dissociation of the vibrationally hot A^+^ ions.

The experiment was carried out for the four PAH molecules mentioned. As shown in Fig. 2b, decay times of 29±4 fs for naphthalene, 32±4 fs for anthracene, 37±3 fs for pyrene and 55±22 fs for tetracene were measured. Moreover, these measured decay times were independent of the IR intensity and the gas used in the XUV high-harmonic generation process (Supplementary Fig. 3). The latter changed the high-energy cutoff of the XUV spectrum from H17 (26.4 eV) in Xe to H23 (35.6 eV) in Ar. The robustness of the measured decay times to changes in the XUV spectral cutoff can be explained by the XUV absorption cross-section of the PAH molecules, which peaks at 17 eV (reaching 400 Mbarn) and decreases substantially above this photon energy^14^,^15^. Hence, only low-order harmonics (H11 (17.05 eV), H13 (20.15 eV) and H15 (23.25 eV)) will be efficiently absorbed and contribute to the observed mechanism. In addition, the cationic states formed are expected to be very similar for all harmonics, with the excess energy being carried off by the ejected photoelectron. Measurements also showed that the decay times barely vary with the IR intensity (Supplementary Figs 3 and 4), meaning that the IR probe intensity does not affect the measured dynamics, and that the relaxation rates are similar for the highly excited A^+^ ions that can be ionized by one or two IR photons and less excited A^+^ ions that require more IR photons. We note that the production of A^2+^ favours ionization processes via the absorption of a limited number of IR photons, since absorption at higher intensity of a larger number of IR photons leads to more extensive fragmentation, and does not contribute to the A^2+^ signal.

The ionization thresholds of the neutral PAH molecules used are 8.1 eV (naphthalene), 7.4 eV (anthracene), 7.6 eV (pyrene) and 7 eV (tetracene). The double ionization threshold of neutral anthracene is ∼20 eV and can therefore be reached directly by H13, H15 and up, but also by, for example, absorption of H11 plus 2 IR photons. However, the latter requires that following the XUV ionization the ion has to remain in an electronically excited state that is close enough to the ionization threshold to be further ionized. Ultrafast non-adiabatic relaxation leads to energy flow from electronic to vibrational degrees of freedom, and therefore increases the number of IR photons that are needed for producing A^2+^, thus causing the observed time dependence.

### Theoretical model

**Figure 3:**
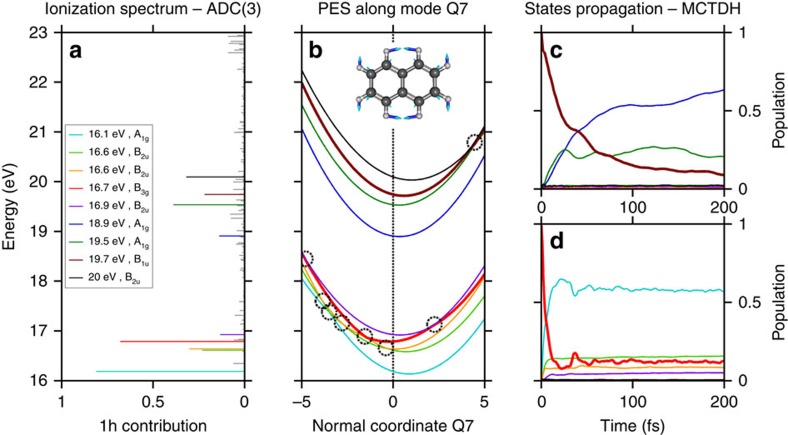
Multi-electronic ADC(3) and non-adiabatic MCTDH propagation calculations performed on naphthalene. (**a**) Zoom of the ADC spectrum in the relevant high energy range, indicating the nine ionic states selected as initial states for the non-adiabatic dynamics simulations (the same colour code indicated in the inset is used in the other parts of the figure). (**b**) Cut through the multidimensional potential energy surface along the normal coordinate of the symmetric inter-ring C=C stretching mode Q7 (see inset), for the nine selected electronic states. The seams (dashed circle) are all located close to the equilibrium geometry (dashed line) and the high complexity of their multidimensional topology governs the depopulation dynamics. (**c**,**d**) Time-dependent evolution of the diabatic population of the electronic states when beginning with population=1 in the B_1u_ (19.7 eV) and B_3g_ (16.7 eV) states, calculated using the MCTDH method.

In order to shed light onto the multi-electronic and coupled electronic and nuclear dynamics, high-level theoretical calculations were carried out (Supplementary Notes 3 and 4). First, a multi-electronic approach was used to compute both the single-ionization spectrum and the multidimensional potential energy surfaces of the PAH molecules, using the third-order *ab initio* algebraic diagrammatic construction (ADC(3)) Green's function method^16^,^17^. Nineteeen ADC calculations were performed for each selected normal mode (corresponding to 19 geometries). This calculation was followed by wave packet propagation employing the multiconfigurational time-dependent Hartree (MCTDH)^18^ approach, to compute the non-adiabatic relaxation of the states determined by the ADC method. These theoretical studies were confined to naphthalene because of the huge computational effort required. In Fig. 3a, we show the high-energy part of the calculated ionization spectrum of naphthalene at 23 eV (Supplementary Fig. 5). Each line represents a cationic eigenstate and is located at the corresponding ionization energy. The height of the line reflects the contribution of a particular one-hole (1h) configuration to the ionic states (accounting for the process of removal of an electron from a particular molecular orbital). Hence, a peak height of 1 reflects a fully mono-electronic process. Reduced peak heights reflect the effect of electron correlation and, more specifically, the involvement of two-hole-one-particle (2h-1p) configurations (accounting for the removal of an electron from a particular orbital accompanied by the excitation of another electron) and higher order configurations. While the ionization below 10 eV is described essentially by 1h configurations, electron correlation effects start to play a very important role above 10 eV, and shake-up excitation and higher order processes dominate above 15 eV (Fig. 3a and Supplementary Fig. 5). Note that a photon with a given energy may populate all states with a lower binding energy (for instance, in our experiment, harmonic H13 populates all states below 20.15 eV). In the experiment, and in agreement with earlier photoelectron spectroscopy measurements^19^, the excitation accesses a spectral domain where correlation cannot be neglected and where excited cations are created due to correlation. The calculation shows that nine states are efficiently populated in the energy range of 16–20 eV (shake-up region) close to the ionization threshold of the cation. This defines the first step of the observed process.

Next, the excited cations relax through non-adiabatic-coupled electron-nuclear dynamics. A model diabatic Hamiltonian^20^ restricted to the nine dominant electronic states (covering the spectral range of interest), calculated using the ADC method described above, was constructed in terms of the dimensionless normal displacement coordinates (Q) of the reference electronic ground state of neutral naphthalene (N) (for technical details, see Supplementary Fig. 6, Supplementary Table 2, Supplementary Notes 3 and 4). In Fig. 3b one-dimensional cuts of the multidimensional diabatic potential energy surface of N^+^ are shown along the normal coordinate of the symmetric inter-ring C=C stretching mode Q7 (ν=1,051 cm^−1^). From this diagram it can be seen that there are numerous curve crossings among this group of states, which develop into multiple multidimensional CIs. The location of the energy minimum of the seam of these CIs with respect to the equilibrium position of a state (Supplementary Table 1) primarily governs the dynamics.

First principles nuclear dynamics calculations were carried out employing the vibronic Hamiltonian constructed as sketched above (Supplementary Note 4). For a selected state, the initial wave packet is defined as a Franck–Condon projection starting from the ground vibronic state of the neutral molecule. The wave packet is propagated over 200 fs in the coupled manifold of 9 N^+^ states with 29 relevant vibrational modes. The 29 chosen modes are the ones that induce noticeable coupling strength in the electronic states according to the symmetry selection rules. Note that the remaining discarded modes have extremely low coupling strength and high frequency and are thus less important in the dynamics. As a typical example, computed time-dependent diabatic electronic populations starting from initial population of the states B_1u_ (19.7 eV) and B_3g_ (16.7 eV) are shown in Fig. 3c,d (the time-dependent populations starting from other states is shown in the Supplementary Fig. 7), and clearly evidence rapid non-adiabatic relaxation dynamics from higher-lying to lower-lying states of the cation. The B_3g_ state (16.7 eV) shows very fast dynamics characterized by a ∼10 fs decay time. The quasi-degeneracy of the seam minima with the equilibrium minimum of this state and moderate interstate coupling causes its ultrafast non-radiative decay. The decay of the B_1u_ state (19.7 eV) occurs within 40 fs, and can again be understood in relation with the location of the seam minima. The discussion above can be generalized to the other electronic states including very low-lying states as presented in the Supplementary Fig. 7 and emphasizes the link between the timescale of the relaxation dynamics and the location of the CIs with respect to the equilibrium position. We note that our model includes only part of all the cationic states present in this energy range. However, as discussed above the decay is determined by topology of the crossings rather than the density of states. The timescale observed is a signature of the rigid bonds of the carbon backbone of the molecule. As a consequence, lower states will only weakly affect the depopulation of states such as B_1u_, as they will lead to similar crossings.

Although modelling of the non-adiabatic relaxation for larger PAH species is computationally out of reach, we expect that the same process will take place in all PAH molecules due to the similarity of their vibrational modes^21^. As shown in Supplementary Fig. 5, the larger PAH molecules have very similar ionization spectra. The relaxation time for larger PAHs is a competition between two effects: on the one hand, the increase in the density of states facilitates rapid energy transfer (the energy gap law). On the other hand, the wave packet has to travel through more intermediate CIs before reaching states from which the system can no longer be ionized by the IR pulse, slowing down the dynamics. This is exactly what we observe experimentally. However, we also note that the evolution of the relaxation lifetime with the size is modest, and even for larger species, it remains in the sub-picosecond regime.

## Discussion

The study reported here involves states at higher energies (typically up to 12 eV above the ground state, that is, binding energy of 20 eV) than those involved in the DIBs (for example, typically below 8 eV above the ground state). Nevertheless, our results also demonstrate that, despite the existence of small energy gaps between the highly excited states probed here, lifetimes in the range of 30–60 fs are observed, and even increasing for large species. Moreover, our calculations show that this decay rate is general and is found for states in the 0–12 eV energy range. This is because of the topology of the CIs. It follows that the energy gap laws cannot be directly extended to large PAHs or higher energy states, suggesting that low-lying states which potentially could be involved in the DIB problem are likely to have much longer lifetimes than previously assumed. As electronic transitions remain the best probe to identify the specific PAH molecules present in space, further studies on the link between PAH cation species and the DIBs are warranted. The use of short XUV pulses to prepare these cation species could have significant implications for unraveling the extent of molecular complexity in the universe.

In conclusion, we have identified for the first time a general process in PAH molecules under conditions where both the single active electron and Born–Oppenheimer approximations breakdown. The XUV ionization is followed by a very fast relaxation of the excited cationic states due to non-Born–Oppenheimer couplings. The present study offers the first direct observation of ultrafast relaxation pathways in a family of large molecular systems under XUV irradiation. The rapid relaxation is due to the favourable topology of the seams of the CIs corresponding to slight distortions of the rigid molecular backbone which relax within a fraction of the vibrational period. The slight evolution of the decay with molecular size indicates that the relaxation remains time confined in the same range even for larger species.

Ultrafast XUV science offers a perfect platform for studying the importance of very short time-scale dynamics in natural processes involving potentially very large molecules. Here we show that, beyond statistical molecular relaxation, other fundamental aspects of quantum mechanics, such as multi-electronic excitation and non-Born–Oppenheimer vibronic couplings play an important role in the context of astrochemistry and have to be accurately included in models. Our work also has possible implications for photoinduced coherent charge transport such as attosecond hole migration following electron removal, as discussed in the recent literature^22^,^23^,^24^,^25^,^26^.

## Additional information

**How to cite this article:** Marciniak, A. *et al.* XUV excitation followed by ultrafast non-adiabatic relaxation in PAH molecules as a femto-astrochemistry experiment. *Nat. Commun.* 6:7909 doi: 10.1038/ncomms8909 (2015).

## Supplementary Material

Supplementary Information Supplementary Figures 1-7, Supplementary Tables 1-2, Supplementary Notes 1-4 and Supplementary References
