# Supplementary material for: XUV excitation followed by ultrafast non-adiabatic relaxation in PAH molecules as a femto-astrochemistry experiment
Source: Nat Commun. 2015 Aug 13;6:7909. doi: 10.1038/ncomms8909 (PMC4557118; doi:10.1038/ncomms8909)
Supplement: Supplementary Information — Supplementary Figures 1-7, Supplementary Tables 1-2, Supplementary Notes 1-4 and Supplementary References [file ncomms8909-s1.pdf]

Supplementary Figures

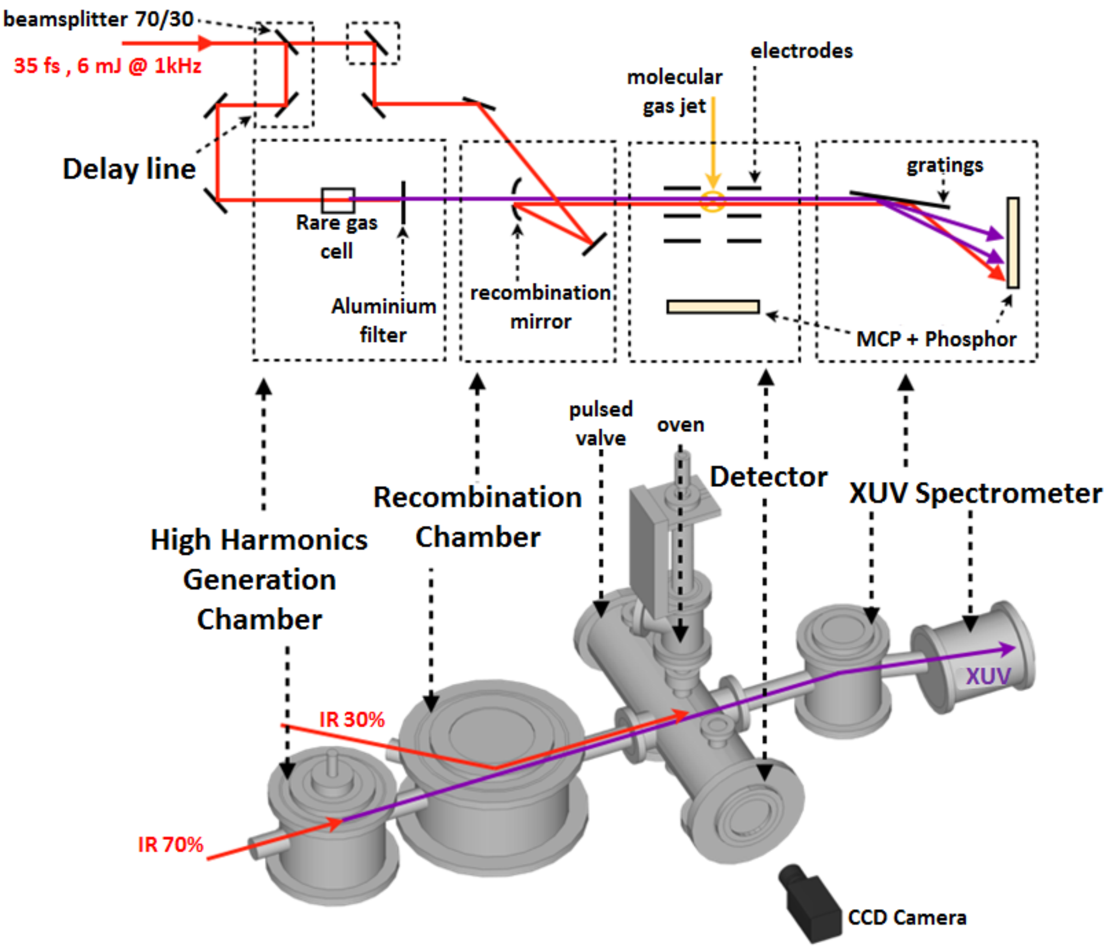

Supplementary Figure 1: Schematic of the pump-probe experimental setup. The experimental setup combines a HHG (High Harmonics Generation) source, a Velocity Map Imaging used in time-of-flight configuration and an oven for the production of the molecular sample.

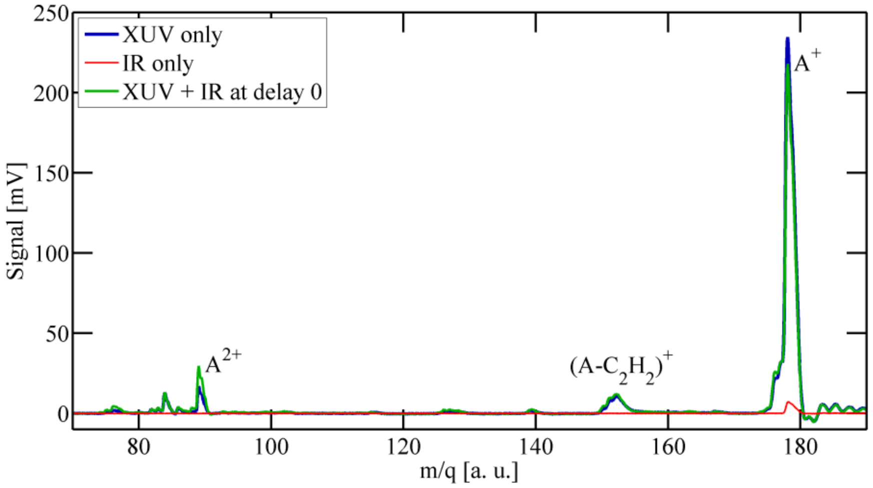

Supplementary Figure 2: Comparison between probe and pump photoion spectra. Time-of-flight photoion spectra of NIR alone taken at I_{IR} = 3.4 10^{11} $W.cm^{-2}$(red), XUV-only taken with HHs generated in Kr (blue) and XUV+IR at delay 0 (green).

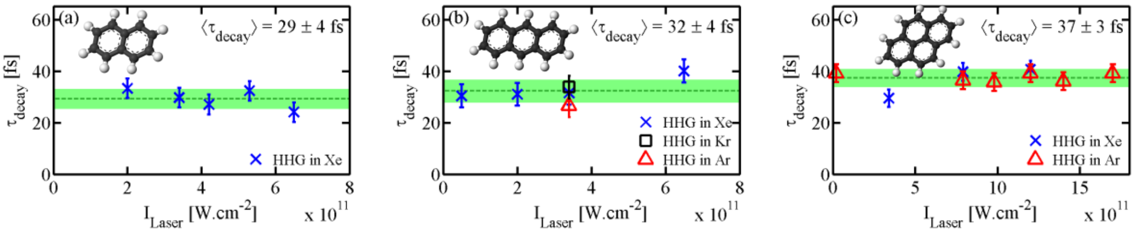

Supplementary Figure 3: Overview of the experimental results. Extracted decay lifetimes for three molecules: naphtalene (a), anthracene (b), pyrene (c) as a function of the IR intensity and for several HHG gas targets. The upper XUV photon energy was 26.4 eV, 32.6 eV and 35.6 eV in the experiments performed with Xe, Kr and Ar, respectively. Each point corresponds to a set of measurements at specific conditions. The error associated to each point is the standard deviation of the complete set of measurements. The dark-green dashed line represents the average value and the green area represents its standard deviation. Within the error bar, the measured decay times are independent on the IR intensity and the HHG gas used.

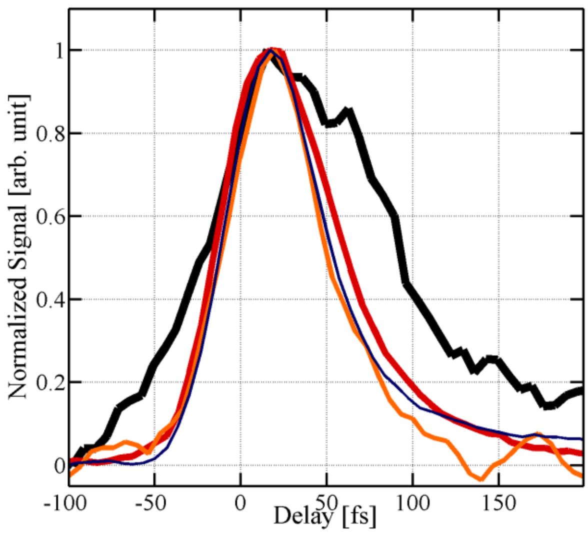

Supplementary Figure 4: Time-dependent dication normalized yield of 4 PAHs. 2D plot of Fig 2.b representing the time-resolved evolution of the naphtalene (blue), anthracene (orange), pyrene (red) and tetracene (black) dication normalized yield. The fitting procedure allows the extraction of the decay time, as well as disentangles the effect of steady states and the cross-correlation of the two pulses.

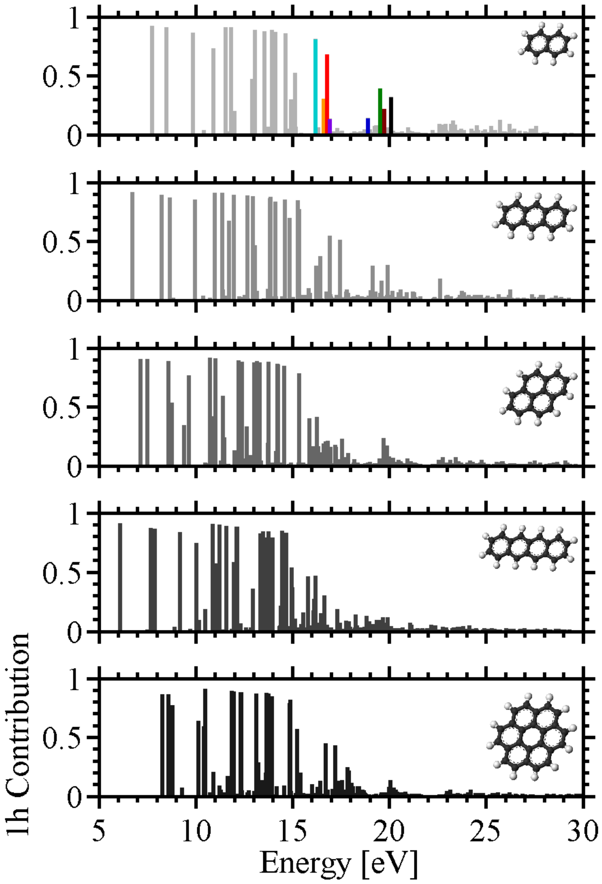

Supplementary Figure 5: ADC spectra computed for 5 PAH molecules from top to bottom: naphthalene (N), anthracene (A), pyrene (P), tetracene (T), coronene (C). For naphthalene, the 9 states used in the model are shown using the same color scale as in Fig. 3 of the article.

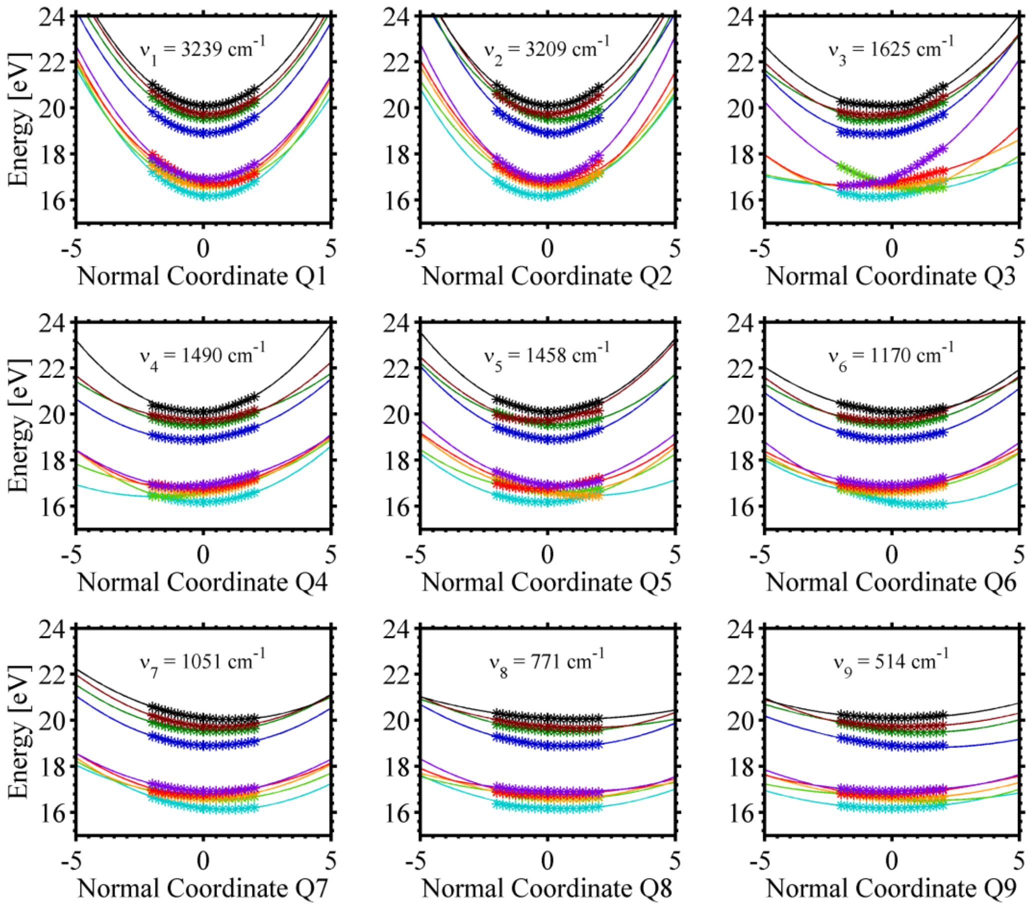

Supplementary Figure 6: One dimensional cuts of the multidimensional potential energy surfaces (PES) of the nine electronic states (see text for details) of N^+ plotted along the dimensionless normal coordinates of the totally symmetric vibrational modes. (The color scale is the one used in Fig. 3 of the article).

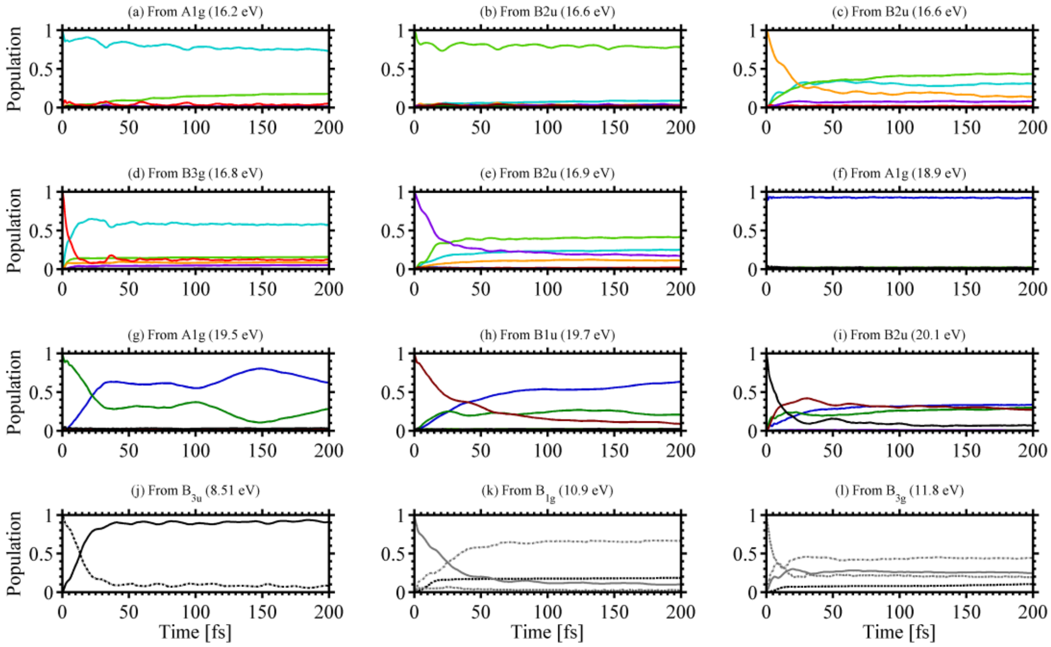

Supplementary Figure 7: Evolution of electronic state population from each initial state. Time evolution of the diabatic electronic populations of the nine electronic states of N^+ calculated by propagating WPs using the MCTDH method and the constructed vibronic model discussed in the text. (a,b,c,d,e,f,g,h,i) correspond respectively to the initially populated electronic states : A_{1g} (16.186 eV), B_{2u} (16.613 eV), B_{2u} (16.636 eV), B_{3g} (16.787 eV), B_{2u} (16.926 eV), A_{1g} (18.907 eV), A_{1g} (19.537 eV), B_{1u} (19.7421 eV) and B_{2u} (20.096 eV). (The color scale is the one used in Fig. 3 of the article).(j,k,l) Low-lying states propagation: B_{3u} (8.5122 eV), B_{1g} (10.5286 eV) and B_{3g} (11.772 eV) (see ref.11 for further details).

Supplementary Tables

State	1	2	3	4	5	6	7	8	9
1		16.27	16.47	16.72	16.41	23.54	26.75	31.15	59.58
2		16.17	16.46	16.65	16.60	20.90	24.01	23.02	24.97
3			16.45	16.64	16.53	25.54	38.39	26.03	36.74
4				16.61	16.63	21.61	24.70	31.24	31.65
5					16.41	22.38	24.32	25.47	30.25
6						18.68	24.62	21.09	23.22
7							19.33	19.57	20.50
8								19.56	20.14
9									19.94

Supplementary Table 1: Energy of minima and seams of the PES. Estimated equilibrium minimum (diagonal entries) and minimum of the seam of various CIs (off-diagonal entries) of the electronic states of N^+. All quantities are given in eV.

Normal modes[a]	SPF basis[b]	Primitive basis[c]
(ν_7, ν_{18}, ν_{32}, ν_{43}, ν_{37}, ν_5)	(8, 10, 8, 10, 12, 10, 8, 12, 10)	[6, 2, 5, 5, 4, 6]
(ν_{36}, ν_6, ν_{30}, ν_{42}, ν_3, ν_{19})	(8, 10, 10, 8, 8, 12, 12, 8, 10)	[5, 7, 4, 5, 6, 4]
(ν_{23}, ν_2, ν_{35}, ν_8, ν_{41}, ν_{29})	(12, 8, 10, 8, 8, 10, 12, 10, 8)	[2, 3, 5, 6, 5, 4]
(ν_4, ν_{31}, ν_{44}, ν_{38}, ν_{40}, ν_1)	(8, 8, 10, 12, 10, 10, 8, 12, 10)	[6, 5, 5, 4, 5, 4]
(ν_{34}, ν_{33}, ν_{22}, ν_{39}, ν_9)	(8, 10, 12, 8, 12, 10, 8, 10, 12)	[5, 5, 4, 5, 8]

[a] Vibrational mode bracketed together were treated as a single particle, e.g., particle 1 is a six dimensional particle including modes ν_7, ν_{18}, ν_{32}, ν_{43}, ν_{37}, ν_5.

[b] The SPF basis is the number of single-particle functions used.

[c] The Primitive basis is the number of harmonic oscillator DVR functions, in the dimensionless coordinate system required to represent the system dynamics along the relevant mode. The primitive basis for each particle is the product of one-dimensional bases.

Supplementary Table 2: Characteristics for MCTDH calculations. Normal mode combinations and sizes of the primitive and single particle basis functions used in the converged wave packet calculations using the MCTDH method in the coupled electronic states of N^+.

Supplementary Note 1: Experimental setup

The experimental arrangement (see Supplementary Fig. 1) consists of a two-color extreme ultraviolet (XUV) plus near infrared (NIR) Mach-Zehnder-type interferometer, a mass spectrometric detection system and an oven for the generation of the molecular beam. A NIR laser beam (800 nm, 35 fs full half width maximum (FWHM), delivering 6 mJ at 1 kHz) was split into two beams. One arm contained the NIR probe beam (with a maximum pulse energy around 1.2 mJ), whose intensity was tuned by changing the size of an aperture. Throughout the experiment, the aperture was adjusted to keep the NIR intensity in the interaction region around 10^{11} $W.cm^{-2}$. An XUV frequency comb was generated by focusing the NIR beam in the second arm into a gas cell containing rare gas such as xenon (Xe), krypton (Kr) or argon (Ar). An aluminum thin filter (300 nm thickness) was used to spectrally filter out the NIR fundamental beam as well as low-order harmonics (below H11). The XUV pump beam was recombined with the NIR probe beam using a drilled mirror. Both the XUV pump and NIR probe beam were collinearly focused onto the molecular target by a toroidal mirror.

The pump-probe experiment was carried out in the active region of a velocity map imaging spectrometer (VMIS) that was used both as a time-of-flight spectrometer and as an electron spectrometer (for laser beam alignment and laser pulse characterization purposes). The molecular sample (naphthalene, anthracene, pyrene or tetracene) was injected into the spectrometer by a heated pipe connected to an oven that sublimates the sample at respectively 30°C, 95°C, 85°C and 210°C. The ion time-of-flight yield resulting from the interaction between the molecular beam and the laser pulses was measured as a function of the pump-probe delay using a 500 MHz oscilloscope (Lecroy waverunner 6050) controlled by a home-made Labview software that was also used to control the delay line. For each pump-probe delay, an ion time-of-flight spectra was recorded by averaging over 20000 laser shots.

We first determined the ionization and fragmentation processes induced by the XUV or the NIR pulse alone (Supplementary Fig 2.a-b). The XUV pulse mainly ionizes the parent molecule M leading to the formation of singly and doubly charged ions M^+ and M^{2+}, and only weakly fragments the molecule through proton loss ($(M\text{-}H)^+$, $(M\text{-}2H)^+$) or loss of larger fragments $(M\text{-}C_2H_2)^+$. The IR pulse leads to single ionization only at the highest laser intensity. (see Supplementary Fig. 2)

Supplementary Note 2: Numerical analysis

The two-color ion time-of-flight signal was recorded, and for each delay the contributions from the XUV and NIR signal alone were subtracted leading to the final signal ΔS (see Eq. 1 main article and Fig. 1).

Here we summarize the method used for data analysis to extract the decay time and the accuracy (Supplementary Fig. 3). The set of data is composed by many measurements to prove the robustness of measured decay time constant with respect to probe intensity, harmonic spectra, cross-correlation duration etc…

For each scan, the formula used to extract the decay lifetime from the di-cation signal is:

$$\Delta S_{Fit,M2+} = \exp(-4\ln(2)(t/\tau_{crossco})^2) \otimes \text{Heaviside}(t-t_0) * [\, A_{Fit} * \exp(-(t-t_0)/\tau_{decay}) + A_{step}] \qquad (II.1)$$

where t_0, τ_{decay} , $\tau_{crossco}$, A_{FIt} are free parameters that respectively represent the zero delay, the decay time, the cross-correlation duration and the magnitude of the signal. A_{step} represents the amplitude of a step function that describes the difference of the baseline at long delays before and after the temporal overlap: it accounts for possible very long lifetimes (steady) states not investigated in this study. Note that for anthracene no step was necessary, hence A_{step} was equal to 0 as discussed in the manuscript.

To de-correlate the free parameters and reduce the error coming from the fit, we used a multi-dimensional fitting procedure based on the one described in the appendix of ref.1. A similar method was used by Stolow et al. in ref.2. The fitting procedure takes into account both the two-color signal from the di-cation and other ions and fragments in the time-of-flight. A de-correlation of the zero delay and the cross-correlation time provides an independent estimate of the decay time. For each scan, the errors coming from the fit correlate with the signal-to-noise ratio and are reasonably small: around 0.9 fs for naphthalene, 3.7 fs for anthracene, 1.5 fs for pyrene.

The decay time constant extracted is defined as the central value of a statistical set of independent measurements. The associated error bar is given by the dispersion of the experimental results. We include in the statistics the measurements performed at different probe intensities and with different XUV spectra (see Supplementary Fig. 3) since no noticeable changes in the dynamics was observed when making these changes to the experiment.

These fitting procedures have confirmed that the timescale of the observed mechanism is independent of the light parameters employed in the experiment (IR intensity, XUV spectrum) within the error bars. The experiment was repeated with four PAH molecules (naphthalene, anthracene, pyrene, tetracene) in order to investigate the generality of the process and its dependence on the molecular size. The ultrafast decay increases slightly with molecular size (Supplementary Fig. 4). For tetracene the decay occurs within 55 ± 22 fs, it takes around 37 ± 3 fs for pyrene, 32 ± 4 fs for anthracene and 29 ± 4 fs for naphthalene (see Supplementary Fig. 3 and 4).

Finally, concerning the fitting procedure used in Fig 2.a on the parent ion, the formula was:

$$\Delta S_{Fit,Parent\ ion} = \exp(-4\ln(2)(t/\tau_{crossco})^2 \otimes \text{Heaviside}(t-t_0) * [\, A_{Fit,decay} \exp(-(t-t_0)/\tau_{decay}) + A_{Fit,diss}(1-\exp(-(t- t_0)/\tau_{diss}))\,] \qquad (II.2)$$

Where $A_{fit,diss}$ is the amplitude of the dissociation process and τ_{diss} represents the population time of the dissociative channels. We extracted the population of dissociating states at about 150 fs.

Supplementary Note 3: ADC(n)

The ionization spectra and the potential energy surfaces of the excited ionic states along the normal modes used in the wave-packet dynamics simulations were obtained using the *algebraic diagrammatic construction* (ADC) scheme[3, 4] of the one-particle many-body Green's function. The ADC schemes are based on perturbation expansion of the Hamiltonian which can be directly compared to the diagrammatic series of the Green's function. A point to stress in this context is that the perturbative character of the method applies only to the ground state of the system and *not* to the final states which are treated non-perturbatively. The calculations were performed with the non-Dyson ADC(3) method[5], being exact up to third order of perturbation theory. This version of the ADC methods uses the so-called *intermediate state representation*[6] (ISR) of the secular matrix, yielding the construction of a complete basis through successive Gram-Schmidt orthogonalization of different classes of correlated excitations.

All calculations were done using cc-pVDZ basis sets[7]. The spectra computed for the 4 PAH molecules (see Supplementary Fig. 5) show similar trends: below 10 eV mainly 1 hole (1h) states are important and above 10 eV, electron correlation cannot be neglected. Above 15 eV shake-up states dominate the spectrum.

Supplementary Note 4: Electron-nuclear Dynamics

A model diabatic Hamiltonian of the nine electronic states calculated using the ADC method described above, corresponding to the main ionization channels that contribute in our experiment, was constructed in terms of the dimensionless normal displacement coordinates (**Q**) of the reference electronic ground state of neutral naphthalene (N). The well-known concept of vibronic coupling theory[8] and elementary symmetry selection rules are utilized for this purpose. Such a Hamiltonian can be symbolically written as

$$\mathcal{H} = (\mathcal{T}_{\mathcal{N}} + \mathcal{V}_0)\mathbb{I}_9 + \begin{pmatrix} W_{11} & \cdots & W_{19} \\ \vdots & \ddots & \vdots \\ h.c & \cdots & W_{99} \end{pmatrix} \quad \text{(IV.1)}$$

Where $\mathbb{I}_9$ is a 9×9 unit matrix and, $(\mathcal{T}_{\mathcal{N}} + \mathcal{V}_0)$, represents the Hamiltonian of the unperturbed reference electronic ground state of the neutral N molecule. The nuclear motion in this reference state is treated as a harmonic oscillator. Accordingly $\mathcal{T}_{\mathcal{N}}$ and $\mathcal{V}_0$ are given by

$$\mathcal{T}_{\mathcal{N}} = -\frac{1}{2}\sum_i \omega_i \frac{\partial^2}{\partial {Q_i}^2} \quad \text{(IV.2)}$$

$$V_0 = \frac{1}{2}\sum_i \omega_i Q_i^2 \qquad \text{(IV.3)}$$

where ω_i represents the harmonic frequency of the vibrational mode i. The matrix Hamiltonian (with elements W) in Eq (1) represents the diabatic energies of the nine electronic states (diagonal elements) of the naphthalene radical cation (N^+) and their coupling energies (off-diagonal elements W_{ij}). The chosen nine doublet states of N^+ are identified by numbers (1 to 9) and they belong to A_{1g} (16.186 eV), B_{2u} (16.613 eV), B_{2u} (16.636 eV), B_{3g} (16.787 eV), B_{2u} (16.926 eV), A_{1g} (18.907 eV), A_{1g} (19.537 eV), B_{1u} (19.7421 eV) and B_{2u} (20.096 eV) electronic terms, respectively, at the equilibrium configuration of the reference electronic state of D_{2h} symmetry (the quantity in the parentheses represents the vertical ionization energy). Using standard vibronic coupling theory, the elements of this Hamiltonian are expanded in a Taylor series around the equilibrium geometry of the reference state at (**Q**=0) as

$$W_{jj} = E_0^j + \sum_{i \in a_g} \kappa_i^{(j)} Q_i + \frac{1}{2}\sum_{i \in all} \gamma_i^{(j)} Q_i^2 \; ; \; j \in 1,2,\ldots,9 \,, \qquad \text{(IV.4)}$$

$$W_{jk} = \sum_i \lambda_i^{(jk)} Q_i \quad ; \quad jk \in 12,13,14,\ldots,89 \; (j \neq k). \qquad \text{(IV.5)}$$

In the above equations, E_0^j represents the vertical ionization energy of the jth electronic state. $\kappa_i^{(j)}$ and $\gamma_i^{(j)}$ are the linear and second-order coupling parameters of the ith vibrational mode in the jth electronic state. The quantity $\lambda_i^{(jk)}$ describes the first order coupling parameter between the j and k electronic states through the coupling vibrational mode i of appropriate symmetry. Therefore, both first and second derivative terms are used in the vibronic model. The parameters introduced in the diabatic electronic Hamiltonian matrix (cf. eq. IV.1) are derived by fitting the calculated *ab initio* ADC electronic potential energies by a non-linear least squares method.

The nuclear dynamics calculations are performed by propagating wave packets (WPs) in the coupled manifold of electronic states employing the multi-configuration time-dependent Hartree (MCTDH) method[9] to numerically solve the time-dependent Schrödinger equation. MCTDH is a grid-based method that utilizes a discrete variable representation (DVR) basis combined with fast Fourier transformation and powerful integration schemes. The efficient multi-set Ansatz of this scheme allows for an effective combination of vibrational degrees of freedom and thereby reduces the dimensionality problem. In this scheme all multi-dimensional quantities are expressed in terms of one-dimensional ones employing the idea of mean-field or Hartree approach. This provides the efficiency of the method by keeping the size of the basis optimally small. Furthermore, multi-dimensional single-particle functions are designed by appropriately choosing the set of system's coordinates so as to reduce the number of particles and hence the computational overhead. The Heidelberg MCTDH suite of programs is employed to propagate WPs in the numerical calculations. For further details of the method and its numerical implementation the readers are referred to ref.10. We included 29 relevant vibrational modes and nine coupled electronic states of N^+ in this study.

In Supplementary Fig. 6 one-dimensional cuts of the multidimensional adiabatic potential energy hypersurface of N^+ are shown along the coordinates of the totally symmetric vibrational modes. In each panel the potential energy values obtained from the present vibronic model and *ab initio* electronic structure calculations are shown by solid lines and points, respectively. It can be seen from Supplementary Fig. 6 that the present theoretical model represents the calculated energies extremely well. From the diagram it can also be seen that there are numerous curve crossings among this group of states. These crossings develop into multiple multi-dimensional conical intersections (CIs). These CIs finally become the mechanistic bottleneck in the nuclear dynamics of a state. The energetic location of the minimum of the seam of these CIs with respect to the equilibrium minimum of a state primarily governs the nuclear dynamics. The location of these energetic minima is calculated within the present theoretical model and the results are given in Supplementary Table 1. In this table the diagonal entries represent the equilibrium minimum of a state whereas the off-diagonal entries represent the energetic minimum of the seam of CIs between states. The importance of these stationary points on the potential energy surface on the nuclear dynamics is discussed below.

First principles nuclear dynamics calculations are carried out employing the vibronic Hamiltonian constructed above. A WP pertinent to the ground vibronic state of the reference state is subjected to a Franck-Condon transition to the excited electronic state of N^+. The Condon approximation of constant transition dipole, well established in a diabatic electronic basis, is used. This vertically promoted wave packet is propagated in the coupled manifold of 9 electronic states of N^+. 29 relevant vibrational degrees of freedom are included in the WP propagation. 9 separate calculations are carried out by initially locating the WP on each of the nine states. The WP is propagated for a total of 200 fs in each case. The numerical details of the converged calculations are given in Supplementary Table 2.

The diabatic electronic populations are recorded in time for each initial excitation and the results are summarized in the Supplementary Fig. 7. In all panels the population on the initially excited state is shown by the solid line. The color scale is the same as the one used in Supplementary Fig. 7. From panel a and b of Supplementary Fig. 7 it can be seen that the decay rate of state 1 and 2 is very slow and about 80% of the population remains in these states at the end of time propagation. Our data reveal that coupling of these states with others is very weak despite the fact that states 1 to 5 are vertically close in energy (vide supra) and although they form energetically low-lying CIs (cf. Supplementary Table 1), they exhibit slow decay. A similar trend of decay can be seen for the state 6 (panel f), this is the slowest decaying state among all. It can be seen from Supplementary Table 1 that the equilibrium minimum of this state occurring at ~18.68 eV is far below the energetic minimum of the seam of CIs of this state with the others. Therefore, this state appears to be very long-lived.

Contrary to the above scenario, the dynamics of states 3, 4, 5, 7, 8 and 9 shown in panels c, d, e, g, h and i, respectively, is faster. For instance, state 4 (panel d) has a life time of about 5 fs. The coupling of this state with state 1 is fairly moderate and six vibrational modes of b_{3g} symmetry participate in it. Its coupling with states 2 and 3 through vibrational modes of b_{1u} symmetry is relatively weak. Now, it can be seen from Supplementary Table 1 that the equilibrium minimum of state 4 occurs only ~ 0.11, 0.04 and 0.03 eV below the minimum of the 1-4, 2-4 and 3-4 CIs. This quasi-degeneracy of the seam minima with the equilibrium

minimum of state 4 and moderate interstate coupling causes its ultrafast non-radiative decay. Decay lifetimes of a few fs are also observed for the highest energy state (cf. panel i, state 9). As it can be seen from Supplementary Fig. 7, the decay of this state is slightly slower compared to state 4, which is again in accordance with the energy data provided in Table 1. The initial decay of this state relates to a time of ~10 fs. A similar analysis reveals that the decay lifetime of the state 3 (panel c), 5 (panel e), 7 (panel g) and 8 (panel h) is ~20, 25, 30 and 40 fs respectively. Therefore, the manifold of the states studied above decays within less than 50 fs as observed in the experiment and in previous theoretical study[11].

Supplementary References

[1] Gitzinger, G., Corrales, M. E., Loriot, V., de Nalda, R. & Bañares, L. A femtosecond velocity map imaging study on *B*-band predissociation in CH_3I. II. The 2^1_0 and 3^1_0 vibronic levels. *J. Chem. Phys.* **136,** 074303 (2012).

[2] Bisgaard, CZ., Clarkin, OJ., Wu, G., Lee, AM., Gessner, O., Hayden CC. & Stolow, A. Time-resolved molecular frame dynamics of fixed-in-Space CS2 molecules. *Science*, **323,** 1464 (2009).

[3] Schirmer, J., Cederbaum, L. S. & Walter, O. New approach to the one-particle Green's function for finite Fermi systems. *Phys. Rev. A* **28,** 1237 (1983).

[4] Cederbaum, L.S. in *Encyclopedia of Computational Chemistry*, edited by P. von Ragué Schleyer, P. R. Schreiner, H. F. Schaefer III, W. L. Jorgensen, W. Thiel, and R. C. Glen, **Vol. 1,** p.1202 (Wiley, Chichester, 1998).

[5] Schirmer, J., Trofimov, A. B. & Stelter, G. A non−Dyson third−order approximation scheme for the electron propagator. *J. Chem. Phys.* **109,** 4734 (1998).

[6] Schirmer, J. Closed−form intermediate representations of many−body propagators and resolvent matrices. *Phys. Rev. A* **43,** 4647-4659 (1991).

[7] Dunning Jr., T.H. Gaussian basis sets for use in correlated molecular calculations. I. The atoms boron through neon and hydrogen. *J. Chem. Phys.* **90,** 1007 (1989).

[8] Köppel, H., Domcke, W. & Cederbaum, L. S. Multi-mode molecular dynamics beyond the Born-Oppenheimer approximation. *Adv. Chem. Phys.* **57,** 59 (1984).

[9] Meyer, H.-D., Manthe, U. & Cederbaum, L. S. The multi-configurational time-dependent Hartree approach. *Chem. Phys. Lett.* **165,** 73 (1990).

[10] Beck, M. H., Jäckle, A., Worth, G. A. & Meyer, H.-D. The multiconfiguration time-dependent Hartree (MCTDH) method: a highly efficient algorithm for propagating wavepackets. *Phys. Rep.* **324,** 1 (2000).

[11] Ghanta, G., Sivaranjana Reddy, V. & Mahapatra, S. Theoretical study of the electronically excited radical cations of naphthalene and anthracene as archetypal models for astrophysical observations. Part II. Dynamics consequences. *Phys. Chem. Chem. Phys.* **13,** 14531-14541 (2011).
